# Supplementary material for: Can we speak of a negative psychological tetrad in sports? A probabilistic Bayesian study on competitive sailing
Source: PLoS One. 2022 Aug 11;17(8):e0272550. doi: 10.1371/journal.pone.0272550 (PMC9371297; doi:10.1371/journal.pone.0272550)
Supplement: Attachment — Submitted filename: Reviewer 2.docx [file pone.0272550.s001.docx]

Reviewer 2

1. It is possible that the authors have more information about the sample that they can show in the work, for example, the years of experience of the athletes or specify the level of the competitions (since it says they are "semi-professionals" but they can only compete at the national level or also internationally, and perhaps the fear of error or burnout can be better understood with this information).

**Response**: The only information we have about the participants in the study is that they are sailors in the 49r class, their age and where they practice sport. Therefore, we have added the following in the limitations section:

Finally, it would be useful to have more demographic or sporting characteristics of the participants in the study.

2. It may also be interesting to indicate at what time of the season the data was taken (whether in preseason, during competitions, etc.).

**Response:** The questionnaire was administered during the season under the supervision of a member of the research group, who explained and answered questions as they were completed.

3. Please, also check some quotes where there are small errors such as indicating the author's initial (for example, line 353) or missing a dot after "et al" (for example, lines 106, 300, 366).

**Response:** We have corrected it.
